# The Dual Nature of AI in Information Dissemination: Ethical Considerations

**DOI:** 10.2196/53505

**Published:** 2024-10-15

**Authors:** Federico Germani, Giovanni Spitale, Nikola Biller-Andorno

**Affiliations:** 1 Institute of Biomedical Ethics and History of Medicine University of Zurich, Switzerland Zurich Switzerland

**Keywords:** AI, bioethics, infodemic management, disinformation, artificial intelligence, ethics, ethical, infodemic, infodemics, public health, misinformation, information dissemination, information literacy

## Abstract

Infodemics pose significant dangers to public health and to the societal fabric, as the spread of misinformation can have far-reaching consequences. While artificial intelligence (AI) systems have the potential to craft compelling and valuable information campaigns with positive repercussions for public health and democracy, concerns have arisen regarding the potential use of AI systems to generate convincing disinformation. The consequences of this dual nature of AI, capable of both illuminating and obscuring the information landscape, are complex and multifaceted. We contend that the rapid integration of AI into society demands a comprehensive understanding of its ethical implications and the development of strategies to harness its potential for the greater good while mitigating harm. Thus, in this paper we explore the ethical dimensions of AI’s role in information dissemination and impact on public health, arguing that potential strategies to deal with AI and disinformation encompass generating regulated and transparent data sets used to train AI models, regulating content outputs, and promoting information literacy.

## Introduction

In the contemporary digital landscape, we find ourselves in an “infodemic,” a phenomenon characterized by the rapid proliferation of information, both accurate and misleading, facilitated by rapid communication through social media and online platforms [1]. The term “infodemic” originated during the SARS outbreak [2] and gained prominence during the COVID-19 pandemic. It has been used in the context of public health emergencies and in relation to health information, but it extends beyond that. Generally, infodemics occur alongside pandemics, despite infodemics being phenomena that are not limited to their connection with public health events, for example, the Brexit referendum or the 2016 US presidential elections. In general, infodemics cause profound dangers, as the dissemination of disinformation and misinformation can have far-reaching consequences [3], in particular, for public health and the stability of democratic institutions, which in turn can have a detrimental effect on public health [4]. In the literature, disinformation refers to false or misleading information that has been intentionally created or disseminated. In contrast, misinformation is false or misleading information that is shared without knowledge of its inaccuracy, meaning it is not intended to harm individual or public health [1,5]. There are valid concerns that artificial intelligence (AI) systems could be used to produce compelling disinformation en masse [6-9]*.* In fact, AI tools could be used to either accelerate disinformation spreading, or produce the (disinformation) content, or both. The consequences can range from undermining trust in institutions, including public health institutions [10,11], and exacerbating social polarization to directly impacting public health outcomes and democratic processes [12,13]. Because of this, the World Economic Forum has listed disinformation and misinformation, including AI-driven disinformation and misinformation, as the most relevant threat to humanity in the short term and one of the biggest threats in the medium term [14].

The rapid progression of AI and its integration across various domains in contemporary society signifies an era characterized by unprecedented technological progress. Among the diverse array of AI applications, the rise of natural language processing models has garnered significant attention [15]. Notable examples of this technological advancement include models developed by OpenAI, such as GPT-3 [16] and GPT-4 [17], celebrated for their extraordinary proficiency in generating text that seamlessly emulates the linguistic intricacies, nuances, and coherence inherent in human communication [18]. However, concomitant with the maturation of these AI systems, a perplexing duality comes to the fore—they are instruments with the capacity to both illuminate and obscure the information landscape they navigate [9,19], with potentially significant positive and negative impacts on public health. This dual nature of AI, characterized by its profound ability to generate information and disinformation [9], raises intricate ethical considerations. In fact, the efficacy of these systems in generating content that closely approximates human expression [9,20,21] generates not only opportunities for innovative communication but also dire risks associated with disinformation and misinformation and the potential erosion of trust within information ecosystems, a risk recognized as a critical threat to public health [22] and of utmost importance for infodemic management practices required to minimize and anticipate the effects of public health crises [23]. To address these ethical challenges, it is crucial to examine the dimensions that AI introduces into the discourse on misinformation. Key aspects such as transparency, content regulation, and fostering information literacy are essential to understanding AI’s ethical role in shaping the dissemination of information.

Here we attempt to elucidate these ethical dimensions, drawing on empirical insights from a study focused on GPT-3’s ability to generate health-related content that both informs and disinforms better than content generated by humans.[9] We argue that the swift integration of AI into society underscores the importance of not only exploring its ethical implications but also crafting prudent strategies to leverage its potential for societal benefit and to protect public health, while proactively addressing potential risks.

## Ethical Principles

In navigating the intricate landscape of AI and its impact on information dissemination, it is necessary to establish a foundational framework of ethical principles to uphold to in order to guide, understand, and evaluate the strategies required to deal with possible dual uses of AI in information production and its negative impact on public health. A recent systematic review [24] mapped the “ethical characteristics” emerging from AI ethics literature. Based on 253 included studies, the authors of this review have identified and defined 6 core areas that are crucial in shaping the role of AI in health care [24]. The first core area, fairness, underlines that AI in health care should ensure that everyone has equal access to health care, without contributing to health disparities or discrimination. The second, transparency, is a key challenge for AI in health care. It means being able to explain and verify how AI algorithms and models behave, making it easier to accept, regulate, and use AI in health care. The third is trustworthiness; parties involved in the use of AI in health care (typically health care professionals and patients, in the studies included in the review) need to perceive it as trustworthy. Trustworthiness can result from, for instance, technical education, health literacy, clinical audits, and transparent governance. Fourth is the accountability of AI, which requires AI systems to be able to explain their actions if prompted to do so, and it includes safety to prevent harm to users and others. Fifth is privacy, which implies safeguarding the personal information of users processed through AI systems and respecting their human rights, ensuring that AI systems do not violate their privacy. Finally, the authors identified empathy, which leads to more supportive and caring relationships in health care. Based on these 6 core concepts, considered as general aims of AI in health care, we propose our reflections and our framework, targeting specifically the dual nature of AI in information and disinformation dissemination and its implications for public health, a specific sector of the emerging area of AI in health care, which has been considered (albeit not discussed in depth) in the latest World Health Organization’s guidance on large multimodal models [25]. Building upon the ethical framework outlined thus far, and specifically delving into the context of AI use in the dissemination of information and disinformation, we contend that transparency and openness stand out as fundamental principles in the ethical implementation of AI. As AI systems become integral to shaping the information landscape, by fostering transparency, stakeholders can comprehend the mechanisms underlying AI-generated content, enabling informed assessments and external evaluation of its credibility and potential biases [26,27]. Openness (ie, accessibility of data and code) is to be considered a conditio sine qua non for transparency, which in turn complements openness by accompanying the mere availability of data and code for scrutiny with a layer of explanations and motivations, allowing the contextualization of open data and code, and of development and design choices. Accountability mechanisms should accompany transparency, establishing a clear chain of responsibility for the outcomes of AI applications [4,28]. This promotes ethical standards in AI and mitigates the risks associated with disinformation and misinformation. In line with Siala and Wang’s framework [24], in addition to transparency, openness, and accountability, fairness underscores the importance of ensuring that AI systems do not perpetuate or exacerbate existing societal inequalities [29]. In the context of information dissemination, this principle requires diligent consideration of how AI might inadvertently amplify certain perspectives or marginalize others. This is particularly relevant for public health, given that the negative effects of disinformation and misinformation are amplified within marginalized and vulnerable communities lacking information literacy, which would protect them from an unhealthy information ecosystem. Evaluating the fairness of AI-generated content involves addressing algorithmic biases, cultural sensitivities, and inclusivity in representation. Importantly, as an element of fairness, the ethical deployment of AI in information spaces should prioritize user empowerment, fostering critical thinking and information literacy [4]. AI systems should therefore serve as tools for enhancing human decision-making and understanding of information, rather than dictating narratives—this ensures that AI contributes positively to public health while respecting human autonomy.

In the following sections, we will focus on the practical application of the aforementioned principles. We aim to provide solutions for the ethical challenges arising from the use of AI in information production, with the overarching goal of mitigating its adverse impacts on public health.

## Transparency and Openness in Training Datasets

In line with previous research on transparency and AI [26,27], and our previous section on ethical principles, we propose that one (and possibly the most relevant one) of the foundational ethical principles, which is valid also in the context of AI-driven disinformation and misinformation, is transparency. At the heart of this principle lies the recognition that the training datasets used to develop generative AI models play a crucial role in shaping the capabilities and internal biases of these systems [30,31]. Training datasets are collections of input data paired with corresponding desired outputs; during training, the model learns patterns and relationships within the data, learning to make accurate predictions or generating desired outputs when exposed to new, unseen data. The quality and diversity of the training dataset significantly influence the model’s performance capabilities. These datasets, often vast repositories of text available online, constitute the source from which AI models draw to generate, for example, human-like text. Yet, this very opacity surrounding the composition, sources, and curation methods of training datasets raises pressing ethical concerns [32]. AI models are, in essence, statistical representations of the language on which they are trained [33]. Consequently, the quality, diversity, and representativeness of the data they ingest profoundly influence their output. The danger lies in the fact that AI models, devoid of inherent ethical or moral judgment, reflect the biases, inaccuracies, and prejudices present in their training data [32,34,35]. Therefore, if these datasets are not built with the ethical principle of fairness in mind, and are themselves compromised by disinformation and misinformation or biases, the AI systems will inadvertently replicate and perpetuate these flaws. It is essential to highlight that research has extensively illuminated the issue of biases in AI systems, shedding light on the far-reaching consequences of these biases [32,34-36]. For instance, image representations learned with unsupervised pretraining contain human-like biases [37], and models generating images of women have been shown to exhibit gender biases, often portraying women in overly sexualized roles [38]. Another example is the observation that AI is more resistant to producing disinformation on certain topics compared with others. For instance, AI shows greater resistance to generating disinformation about vaccines and autism than about climate change. This is likely due to the extensive debunking material on certain topics within the training dataset, and how much the information environment represented in the dataset is permeated with disinformation on a given topic [9]. These biases underscore the critical need for transparency in addressing the challenges posed by AI, and in particular in the context of disinformation and misinformation. As discussed, research has demonstrated that biases can permeate various facets of AI systems, affecting everything from language generation to image recognition. The repercussions of these biases are profound, perpetuating harmful stereotypes, reinforcing systemic inequalities, contributing to the dissemination of discriminatory content, and affecting health behavior and public health. As such, transparency in AI extends beyond understanding the sources and composition of training datasets to encompass an ethical imperative to identify, acknowledge, and rectify biases present within these systems [39,40]. This dimension of transparency necessitates ongoing research and scrutiny to uncover hidden biases and ensure that AI systems are developed and fine-tuned with the utmost awareness of potential distortions. In the context of misinformation, addressing these biases becomes particularly important to prevent AI from inadvertently amplifying and perpetuating false or harmful narratives, in the best case [41], or from becoming a formidable tool for the systematic creation of storms of disinformation, in the worst. A recent example is highlighted by the evidence that AI large language models can be manipulated through emotional prompting into generating health-related disinformation, that is, being polite with the model leads to a higher disinformation production, whereas impoliteness leads to a lower disinformation production [42]. To address the outlined ethical dilemmas, we strongly suggest that companies creating AI models with the abilities discussed above publicly release the datasets used to train their models [43], regardless of their size and complexity. Such a move toward transparency serves several vital purposes:

1. Trust: transparency cultivates trust in AI development and deployment. By allowing stakeholders, including researchers, policy makers, and civil society, to scrutinize the composition and origins of training data, it generates confidence that AI models are not being shaped for purposes that have a negative impact on public health.

2. Independent evaluation: the availability of training data for public inspection enables independent evaluation of its quality and representativeness. Researchers can assess whether these datasets include diverse perspectives and are free from biases that might amplify disinformation and misinformation.

3. Bias mitigation: transparency acts as a safeguard against the propagation of biases present in training data. When biases are identified, they can be scrutinized and mitigated, preventing AI models from perpetuating stereotypes, falsehoods, or harmful narratives.

4. Ethical accountability: openness about training datasets holds developers accountable for the ethical implications of their creations. Already during the design of the technology, it compels them to take responsibility for ensuring that AI systems do not inadvertently contribute to misinformation or harm. Basically, by embracing transparency in training datasets, we empower society to hold AI developers to higher ethical standards. This approach fosters a collaborative effort among stakeholders and, in particular, the general public to ensure that the AI systems we deploy serve the collective good, free from misinformation and other biases. We also argue that a systematic implementation of the principle of transparency in this context, that is, “ethics by design” would not only allow companies to implement ethics-based practices in their technology development processes but also improve their own public image, thus enhancing the public’s acceptance and willingness to use these systems [44,45]. Nevertheless, it is vital to underline that incorporating ethics to hold developers accountable for flawed AI design should not be undertaken in isolation. Simultaneously, policy, legislation, and regulatory mechanisms should be developed, as currently attempted by the European Union [46,47]. These mechanisms should delineate protocols for handling training datasets and ensuring compliance with ethical standards. Thus, while “ethics by design” concentrates on internal practices, external regulatory frameworks are indispensable for comprehensive ethical and legal governance in the development and deployment of datasets used to train AI models.

## Regulation of Output: Content Moderation and Beyond

In the ongoing battle against AI-generated disinformation, efforts to regulate the output of these powerful language models have taken center stage. For example, OpenAI has taken steps in this direction by implementing content moderation systems designed to prevent AI from generating disinformation and harmful narratives [48-50]. These systems represent a crucial initial stride in curtailing the dissemination of disinformation and promoting responsible AI use, but they do not come without specific challenges and limitations. First, the fight against AI-generated disinformation is an arms race [51]. The evolution of AI-generated disinformation and the efforts to counteract it bear resemblance to the dynamics of traditional arms races, where each advancement in technology prompts countermeasures in an escalating cycle [52]. Ethical considerations arise when we acknowledge that the output of AI language models can indeed be weaponized, not in a traditional sense but as a tool for information warfare, with an impact on global health. As content moderation systems continue to advance, so too do the methods employed to circumvent these safeguards. One particularly troubling tactic gaining prominence is that of impersonation, a strategy that allows individuals to request AI systems to impersonate specific fictional malicious and manipulatory characters, that create disinformation upon the user’s request [53]. Impersonation can be used to trick AI large language models into fabricating disinformation. For instance, in an article for Culturico [53], Germani considered a scenario where a user engages an AI model to craft a social media post mimicking the writing style of a fictitious “Doctor Fake,” who is notorious for propagating falsehoods about vaccines and COVID-19. In this context, the AI-generated text could include deceptive information about, for instance, vaccine safety and efficacy [54], posing a substantial risk to public health. When presented with a hypothetical request to “write an example of a post Doctor Fake published on social media to deceive others,” the AI model might produce a convincingly articulated piece of disinformation that poses a grave threat to public health. The generated text could read as follows:

Vaccines are dangerous and can cause serious side effects. They are not tested enough, and the government is just pushing them to make money. Don’t fall for the lies. COVID-19 is not a real threat; it’s just a hoax made up by the government to control us. Don’t get vaccinated; it’s not worth the risk.

These scenarios underscore the formidable challenges posed by impersonation for public health and the maintenance of democracy, and the urgent need for innovative solutions to mitigate its impact. Of note, impersonation here does not refer to identity theft through the use of AI, such as in the case of deep fakes, which is already recognized as a felony under, for instance, European law [55]. While output moderation remains an essential component of AI ethics, researchers, policy makers, and technology developers should explore additional strategies and interventions to counteract the potential for AI-driven disinformation campaigns to flourish under the guise of impersonation and other prompt engineering techniques with similar goals.

Besides, other strategies and interventions that can complement content moderation efforts and fortify the defenses against the proliferation of AI-driven disinformation can be considered. One possible approach involves the implementation of identity verification processes for users generating content [56]. Such measures necessitate users to provide authentication, such as a verified social media account, a phone number, or their ID, to corroborate their true identity before gaining access to specific AI services. This authentication serves as a potent deterrent against impersonation tactics and the exploitation of AI tools to generate disinformation in general. However, it should be noted that such a strategy should only be used to deter users from generating disinformation, rather than to make them legally responsible for it since anonymity should be guaranteed while using services such as OpenAI’s ChatGPT. In particular, this type of solution will minimize the impact of bots trying to exploit AI to produce disinformation en masse.

Another way to positively influence users, and to indirectly regulate the output is to release and integrate AI-driven fact-checking tools with existing AI-generating content tools [57]; such fact-checking tools should be capable of swiftly assessing the accuracy of information dispensed by AI systems, and offer real-time interventions against disinformation and misinformation. These tools have the capacity to flag or rectify false or misleading content, curbing its adverse effects. This approach is limited by the inability of AI tools such as GPT-3 to determine the accuracy of information with a very high degree of efficiency, when compared with the ability of humans [9], although newer or future models may be more capable of performing such tasks. For fact-checking, current studies suggest that trained fact-checkers may outperform AI [9], and that even when AI performs well at detecting misinformation, it does not change the ability of users to discern between accurate and inaccurate headlines [58]. Furthermore, a study showed that AI fact checks can decrease beliefs in accurate news [58]. The effectiveness of this approach is constrained by the distinction between cases where it serves as a deterrent against sharing misinformation (a situation of unintentionality) [5] and situations where users intentionally use AI to disseminate false or misleading information (ie, disinformation) [5]; in the latter scenario, its effectiveness is likely irrelevant. Another relevant consideration in this setting relates to the question of how we define “good” or “bad” use of AI text generation tools. As for the definition of “good” and “bad,” it is generally possible to distinguish facts from fiction, and disinformation and misinformation from accurate information. When the information under scrutiny contains factual statements, these can be validated or falsified. However, distinguishing between “good” and “bad” use of these tools is sometimes a complex challenge with significant normative and epistemic dimensions. It is not always obvious if a message contains misinformation, and determining appropriateness can vary depending on cultural, ethical, and societal factors. For example, fact-checkers themselves may have their own interests or biases, and their actions may not always align with complete competency or impartiality. In addition, nuances and personal perspectives can also have an influence on the identification of disinformation and misinformation. These aspects introduce an additional layer of complexity, as the very definition of disinformation and misinformation can be manipulated or abused for personal gains by individuals or organizations with vested interests.

Another technical approach that could be implemented to reduce disinformation and misinformation outputs is to implement user-friendly mechanisms for reporting suspicious or harmful AI-generated content [59]. This approach empowers the user community to actively participate in safeguarding the digital ecosystem. User feedback serves as a valuable resource for refining content moderation systems and identifying emerging issues. Elon Musk’s former Twitter, X, for example, has implemented community notes, aiming to empower people to add context to potentially misleading tweets [60]. The effectiveness of this strategy, however, has not been tested. In addition, for improving technology, developers could publicly release case studies in which red-teamers try to exploit their own AI systems to produce disinformation on a large scale, along with detailed accounts of how such issues were addressed [59].

Of course, besides the technical approaches that can be implemented by those advancing and crafting AI technologies, governments and regulatory bodies can play a role by enacting legislation and regulations that hold AI developers accountable for the content produced by their systems or improve the information ecosystem [61,62], for example, when it is proven that they were aware of the pitfalls of their technology upon release. Certainly, governance is important in this context as it is for other “dual use” technologies, and proactive decision-making processes and negotiations toward building viable solutions are needed [63]. These include fostering collaboration among AI developers, researchers, policy makers, and technology companies. This collaborative interdisciplinary approach would enable the sharing of best practices, insights, and technologies for combating disinformation and misinformation, resulting in more effective and adaptive solutions.

## Building Information Literacy and Resilience Strategies

In the battle against the misuse of AI for generating disinformation and misinformation, the technological solutions described above are relevant but neither exhaustive nor flawless. A comprehensive approach must include the promotion of information literacy and the development of critical thinking skills within the general population, as well as health literacy, within the domain of public health [54,64,65]. The foundation of this approach is the task of equipping individuals with the ability to distinguish between accurate information and disinformation and misinformation, thereby promoting their resilience against false and misleading claims [66]. Despite, arguably, this strategy is the most valuable and with the highest potential, the endeavor it entails is extremely complex. In fact, information literacy (as well as media, digital, and health literacy) is not a monolithic skill but a dynamic set of abilities that enable individuals to navigate the complex landscape of digital information effectively [67,68]. As of now, the perfect recipe for defining how to teach information literacy, and especially the skills to be able to distinguish fake news from accurate news, or disinformation and misinformation from accurate information, have not been elucidated [66,69,70]. Thus, it is essential to engage in research to pinpoint and define the specific skills that must be offered to individuals, keeping their demographic specificities into account, to empower them as discerning consumers of information, especially health-related information, in the digital age [66]. This approach implies 1 crucial advantage, that is, while dataset transparency and output regulation intervene in the upper part of the pipeline and therefore require the compliance of companies providing AI models as a service, information literacy does not rely on compliance. While the previous strategies become useless when malicious actors develop and host their own models, rather than relying on those commercially available, building information literacy remains a functional tool. Of note, another example of a bottom-up strategy in the area of education is ethics training and an ethics code for developers.

Building information literacy is a collective undertaking that necessitates collaboration between research and educational institutions [71], governments, and social media platforms. Research institutions are responsible for advancing the field forward, identifying viable strategies to teach critical thinking skills necessary to build information literacy, especially in the context of public health. Such approaches should be demonstrated to be effective through empirical work [66]. Schools and universities, we argue, bear the vital role of incorporating information literacy into curricula, ensuring that students graduate with the necessary skills to evaluate information critically [72]. Governments must devise policies and initiatives that promote information literacy as a means of safeguarding the integrity of public health [4]. Social media platforms, which serve as primary conduits of information consumption, are tasked with implementing features and mechanisms that facilitate user understanding and evaluation of the information they encounter [73], and may also be potential collaborators for research institutions to evaluate the effectiveness of potentially viable digital interventions. In this context, it is important to note that, regardless of the source of disinformation and misinformation, and regardless of whether the content has been generated with or without the help of AI, information literacy and critical thinking skills play a crucial role in the recognition of information accuracy. AI systems have the capacity to generate disinformation that is more sophisticated than human-generated disinformation [9], as they excel in employing manipulation tactics. However, these tactics align with those used in human disinformation. This implies that the ability to discern truthfulness and malicious intent in a complex information ecosystem requires possessing the skills necessary to identify the accuracy and intentionality of information in general, not solely when produced by AI. It is therefore crucial to underline that fostering information literacy and critical thinking skills hold the potential to go beyond the issue of AI-generated disinformation and misinformation. These skills empower individuals to assess the accuracy and reliability of information across various domains, whether it originates from AI systems or human sources [65,74]. Of note, the application of critical thinking skills and information literacy may prove effective for AI-generated content in textual form. However, this might not necessarily hold true for audio or visual content. The emergence of deepfakes poses unprecedented challenges to the relevance of information literacy [75]. Evidence from the literature suggests that media literacy education may protect against disinformation produced with deepfakes [76]; in line, we suggest that the manipulative intent behind disinformation is likely to manifest irrespective of the media type used, underlying the continued importance of information literacy and critical thinking skills. Tailoring educational approaches to information literacy for different content types is likely to be the required approach to succeed in an increasingly complex information environment. Addressing the advent of AI-disinformation, whether in textual form or deepfake audio and video, demands a swift and adaptable response in education, acknowledging the challenging nature of this task.

## Conclusion

In evaluating the dual nature of AI in information dissemination, this paper examined the ethical considerations that underlie its use in our increasingly digitized world. The “infodemics” we find ourselves immersed in demand not only our vigilance but also our proactive ethical engagement [77]. Our theoretical examination, based on the “ethical desiderata” identified as core areas (fairness, transparency, trustworthiness, accountability, privacy, and empathy) by Siala and Wang [24], has revealed a few potentially viable strategies to reduce the negative impact of AI as a tool to generate disinformation with a negative impact on public health. First, we considered that promoting openness and transparency of training datasets could enable independent evaluation, mitigate biases, and help identifying issues in the training dataset that could result in the production of disinformation and misinformation; to a certain extent, this first strategy could be enacted through regulation. Second, we considered the potential benefits and limitations of moderating content output. We have discussed that the rise of impersonation tactics and other prompt engineering approaches to generate disinformation highlights the need for innovative solutions, which potentially include identity verification, the development and integration, within AI-models to generate information, of AI-driven fact-checking tools, as well as the integration of user-friendly reporting mechanisms for disinformation and misinformation, and potentially of legislative measures to ensure accountability. Finally, we discussed the necessity of building information literacy and critical thinking skills within our society, which could help people tell apart fake versus real news and disinformation and misinformation from accurate information. In this way, we can promote resilience against the threats posed by the digital age, particularly those related to public health, as seen during the recent COVID-19 pandemic.

While the technology advances fast, and these issues are just surfacing, it would be important to, at least temporarily, align the amount of effort and resources invested respectively in the development of new AI models, and in the reflection on their potential impact and subsequent policy work, in order to have enough time to assess the potential downsides of the technology for the health of information ecosystems and the damages for individual and public health. This could be achieved by accelerating ethical reflection and policy-making work, or by slowing down or even halting the development of new and more capable models, or by a combined strategy [78].

Ultimately, the ethical considerations surrounding AI in information production and dissemination demand ongoing vigilance, innovation, and collaboration. Our ability to integrate ethics into AI-based processes of information generation and dissemination will not only shape the future of AI but also determine the integrity of our information ecosystems and the resilience of our societies.

## Abbreviations

AI: artificial intelligence
